# Adherence to the Mediterranean Diet and Its Association with the Level of Physical Activity in Fitness Center Users: Croatian-Based Study

**DOI:** 10.3390/nu13114038

**Published:** 2021-11-12

**Authors:** Dinko Martinovic, Daria Tokic, Lovre Martinovic, Marko Kumric, Marino Vilovic, Doris Rusic, Josip Vrdoljak, Ivan Males, Tina Ticinovic Kurir, Slaven Lupi-Ferandin, Josko Bozic

**Affiliations:** 1 Department of Pathophysiology, University of Split School of Medicine, 21000 Split, Croatia; dinko.martinovic@mefst.hr (D.M.); lm71805@mefst.hr (L.M.); marko.kumric@mefst.hr (M.K.); marino.vilovic@mefst.hr (M.V.); josip.vrdoljak@mefst.hr (J.V.); tticinov@mefst.hr (T.T.K.); 2 Department of Anesthesiology and Intensive Care, University Hospital of Split, 21000 Split, Croatia; dtokic@kbsplit.hr; 3 Department of Pharmacy, University of Split School of Medicine, 21000 Split, Croatia; doris.rusic@mefst.hr; 4 Department of Surgery, University Hospital of Split, 21000 Split, Croatia; ivmales@kbsplit.hr; 5 Department of Maxillofacial Surgery, University Hospital of Split, 21000 Split, Croatia; slupif@kbsplit.hr

**Keywords:** Mediterranean diet, physical activity, dietary supplements, fitness, MDSS, IPAQ-SF

## Abstract

The Mediterranean diet (MD) is based on the traditional cuisine of south European countries, and it is considered one of the healthiest dietary patterns worldwide. The promotion of combined MD and physical activity has shown major benefits. However, the association between physical activity and the MD in regular fitness center users is still insufficiently investigated. This cross-sectional survey-based study was conducted on 1220 fitness center users in Croatia. The survey consisted of three parts: general information, the Mediterranean Diet Serving Score (MDSS) and the International Physical Activity Questionnaire Short Form (IPAQ-SF). The results showed that 18.6% of fitness center users were adherent to the MD, and there was a significant positive correlation between the level of physical activity and the MDSS score (r = 0.302, *p* < 0.001). Moreover, after dividing the sample into tertiles based on the IPAQ-SF score, the third tertile (MET > 3150 min/wk) had the most fitness center users (34.4%) adherent to the MD, while the first tertile (MET < 1750 min/wk) had the least (6.1%). These outcomes emphasize the importance of physical activity as they imply that, with higher levels of physical activity, people are also possibly more aware of the importance that a healthy and balanced diet has on their well-being.

## 1. Introduction

It is well established that regular exercise combined with a diversified and balanced diet can lead to a longer, healthy and more satisfying life [1,2,3]. Physical activity as a beneficial mechanism on health was first investigated in 1950s in correlation with cardiovascular diseases [4]. Since then, exercise was thoroughly researched for its beneficial impact on health. In addition to reducing the risk of developing cardiovascular diseases, it was established that physical activity also has a positive impact on preventing serious illnesses such as diabetes, depression and obesity [5,6]. As aforementioned, a healthy diet is as important as exercise, and an adequate intake of macronutrients and micronutrients with proper hydration is the base of a balanced diet. Furthermore, besides a balanced diet, physically active people also commonly use dietary supplements to enhance results and ensure optimal nutrient intake [7]. Dietary supplements can be defined as food additions containing higher levels of proteins, vitamins and other micronutrients made to amplify and boost regular diet [8].

Whereas some of the widespread diets have been developed in cooperation with nutritionists and physicians, some diets are based on the tradition of a specific region or a country [9]. The Mediterranean diet (MD) is based on the traditional cuisine of south European countries on the shores of the Mediterranean Sea. The foundation of this diet is the high intake of olive oil, vegetables, fruits, nuts, cereals and legumes, while the intake of fish, red wine, dairy products and meat should be moderate [10]. However, white meat should be consumed more frequently than red meat. Moreover, individuals should maintain a small, continuous consumption of red wine, and the recommendation for men and women is 1–2 glasses/day and 1 glass/day, respectively. Lastly, there is a low intake of eggs and sweets. The MD has been the interest of numerous studies, which have aimed to elaborate how exactly it affects the health of its consumers [11,12]. Higher adherence to this diet has shown many benefits to health, such as the reduced risk of developing diabetes, obesity, hypertension, cognitive diseases, hyperlipidemia and depression [13,14,15,16,17,18]. A multicenter trial in Spain showed that an MD with a higher intake of olive oil directly reduces risk of cardiovascular incidents, which is also in line with other studies that have shown the protective role of olive oil and the MD in several serious conditions, such as coronary disease and stroke [19,20,21,22,23].

A recent study by Iaccarino Idelson et al. showed that there is an association between physical activity and adherence to the MD, while they also have an inverse correlation with sedentary behavior [24]. Moreover, a systematic review with a meta-analysis by Malakou et al. found that the promotion of combined MD and physical activity showed a significant metabolic risk reduction [25]. In addition, the results of several Spanish studies have implied that the level of physical activity has a significant correlation with adherence to the MD [26,27,28]. However, MD could have some shortcomings in the physically active population, as the study by Passariello et al. has raised the question of whether MD can meet sufficient protein requirements [29]. Nevertheless, the results regarding the adherence to the MD and its connection to the level of physical activity in the population of regular fitness center users are still inconclusive.

Hence, the aim of this study was to investigate the association between physical activity and the adherence to the MD in fitness center users. Moreover, we aimed to evaluate the adherence to the specific dietary components of the MD and to assess the usage of dietary supplements and its association with MD in this population.

## 2. Materials and Methods

### 2.1. Study Design and Ethical Considerations

This cross-sectional survey-based study was performed among fitness center users in Split, Croatia, during the time period from July to October 2021. The restrictions in Croatia due to COVID-19 pandemic were lifted on February 15, and fitness centers have been operated normally since then.

The study was approved by the Ethics Committee of University of Split School of Medicine (No: 003-08/20-03/0005) and was conducted in regulation with the latest Helsinki declaration. The subjects gave consent to participate by submitting a completed questionnaire.

### 2.2. Participants

The study was conducted on fitness center users in Split, Croatia, using an online survey constructed with the Google Forms^®^ application. The survey was distributed among fitness centers users using QR codes and emails, and through closed social media groups and fitness trainers. 

Participation in the study was voluntary, and anonymity of the provided answers was guaranteed. The inclusion criteria were: 18–65 years of age, and using the fitness center for more than 3 months on at least a 1-time-per-week basis. The only exclusion criterion was involvement in professional sports. Professional sports were defined as involvement is all sport activities for which the participant receives payment and/or is competing in the professional tournaments, with the additional requirement that the subject is involved in that sport activity for ≥6 days/week.

### 2.3. Questionnaires

The survey consisted of three parts. The first part was the questionnaire which included general information about the participants such as the gender, age, anthropometric traits, frequency and duration of the training in the fitness center, involvement in professional sport and habits about usage of dietary supplements. The questionnaire included 12 items, and it was developed for the purpose of this study after extensive review of the available literature.

The second part of the survey was the Mediterranean Diet Serving Score (MDSS), a reliable, validated 14-item questionnaire with a verified Croatian version [30,31]. The MDSS is used to assess the adherence to the MD, and it is updated by the latest guidelines of the Mediterranean Diet Pyramid based on the frequency of consuming certain food and food groups and scoring them by one (1), two (2) or three (3) points depending on the recommendation on intake. Fruits, vegetables, cereals and olive oil are scored by three points, meaning they should be consumed every meal. The two-point foods are dairy products and nuts, which are recommended to be consumed daily. Lastly, one point goes to white meat, red meat, fish, potatoes, legumes, eggs, sweets and wine, which should be consumed once a week. The cutoff value for determining adherence to MD is a total MDSS score of ≥14 points.

The third part of the survey was the International Physical Activity Questionnaire Short Form (IPAQ-SF), an open-ended, reliable, validated questionnaire, which was verified in a Croatian version [32,33]. The IPAQ-SF evaluates the self-reported activity of four intensity levels: vigorous-intensity activities, moderate-intensity activities, walking and sitting [28,29,30]. It was suggested by the IPAQ-SF authors that, for observational studies, the “last 7 days recall” version should be used. MET (metabolic equivalent of task) minutes per week scores were calculated from the results of the IPAQ-SF according to the following formulas [34]:

- Walking MET-min/week = 3.3 × walking minutes × walking days
- Moderate MET-min/week = 4.0 × moderate activity minutes × moderate days
- Vigorous MET-min/week = 8.0 × vigorous activity minutes × vigorous days
- Total MET-min/week = walking + moderate + vigorous MET-min/week scores

### 2.4. Survey Pre-Testing

A survey pre-testing was conducted on a sample of 43 randomly chosen fitness center users. The average time needed to complete the survey was 12 min. The feedback from the responders showed that all the questions were clear and understandable. The final version of the survey consisted of 33 questions.

### 2.5. Statistical Analyses

All statistical analyses were performed using MedCalc for Microsoft Windows (MedCalc Software, Ostend, Belgium, version 17.4.1). Normality of distribution was evaluated using the Kolmogorov-Smirnov test. Continuous variables were presented as mean ± standard deviation or median (interquartile range) depending on the distribution normality. Categorical variables were presented as a whole number (N) with percentage (%). For determining differences between the groups, an independent samples t-test was used for continuous variables with normal distribution, whereas the Mann–Whitney U test was used for continuous variables with non-normal distribution. The chi-square (χ^2^) test was used to determine differences between groups in terms of categorical variables. To investigate the correlation between variables, we used Spearman’s rank correlation coefficient. Comparison of parameters between IPAQ-SF tertiles was performed using either one-way analysis of variance (ANOVA) with the post-hoc Tukey test or one-way analysis of variance on ranks with the post-hoc Dunn’s test. A multiple linear regression analysis with a forward algorithm was applied to determine significant and independent correlates of the total MDSS score, which was defined as a dependent continuous variable. From these analyses, we reported the respective *p*-values with unstandardized β-coefficients, standard error and t-values. In addition, the independent predictors for adherence to the MD were evaluated with multivariable logistic regression, with the OR (odds ratio), 95% CI (95% confidence interval) and *p*-value reported. The level of statistical significance was set at *p*-value < 0.05.

## 3. Results

### 3.1. Baseline Characteristics

The study included 1220 participants, and there were 690 (56.5%) male and 530 (43.5%) female fitness center users. Their mean age was 29.1 ± 8.8 years. Most of them (52.6%) had the education level of master’s degree, while the least (0.6%) had only elementary school. Furthermore, most of them (65.8%) were using dietary supplements, out of which whey protein was the most used supplement (76.8%) (Table 1).

In regard to gender differences, male participants had a significantly higher weight (87.2 ± 13.0 vs. 68.9 ± 12.1 kg, *p* < 0.001), height (184.8 ± 7.3 vs. 172.0 ± 7.4 cm, *p* < 0.001) and BMI (25.4 ± 2.9 vs. 23.0 ± 3.6 kg/m^2^, *p* < 0.001), while female participants were significantly older (28.2 ± 7.8 vs. 30.3 ± 9.9 years, *p* < 0.001). Furthermore, a significantly higher number of male participants were using dietary supplements (71.4% vs. 58.5%, *p* < 0.001) (Table 1).

We divided the study sample in tertiles using the IPAQ-SF results. The first tertile consisted of 407 participants, and their total MET min/week was <1750. The second tertile had 406 participants, and total MET min/week was 1750–3150, while the third tertile had 407 participants, and total MET min/week was >3150. There was a significant difference regarding gender in the three groups, as the first tertile had the lowest number of males (47.4%). Moreover, the first tertile had the highest number of high school education-level participant (32.7%), while the third tertile had the highest number of master’s degree-level participants (58.0%). In addition, the third tertile had the highest usage of dietary supplements (73.7%). Out of the three groups, the third tertile also had the highest usage of whey (98.6%), BCAA (57.3%) and creatine (39.6%). Furthermore, the third tertile had the highest portion of participants who used the fitness center for more than 7 years (30.5%) (Table 2).

### 3.2. MDSS Results in the Study Sample

**Figure 1:**
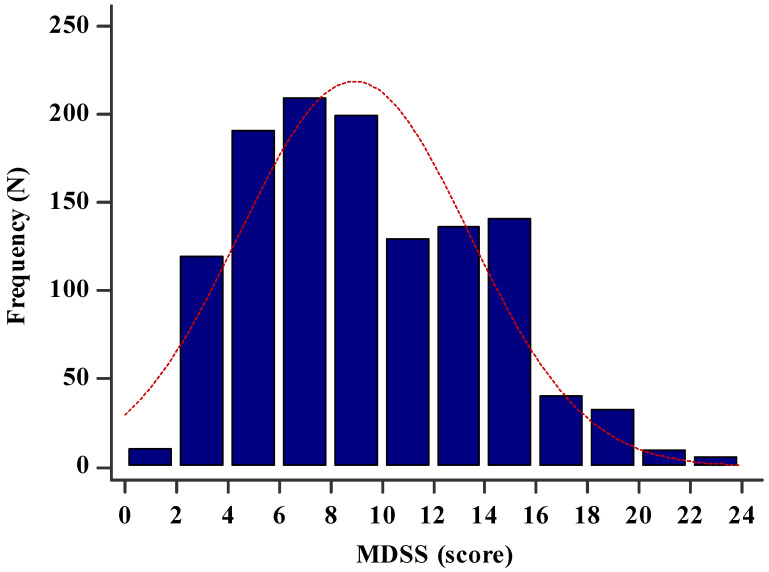
Histogram showing the MDSS score in the study sample (*n* = 1220).

**Figure 2:**
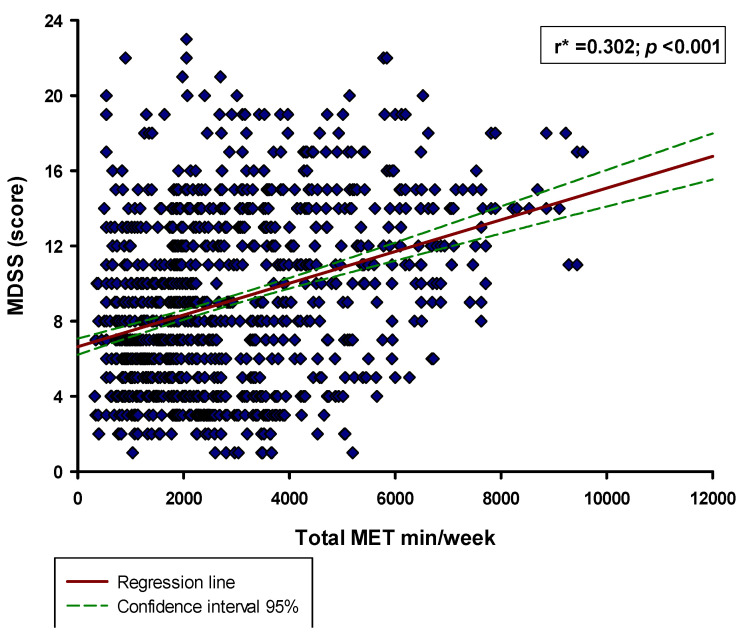
Correlation between the total MDSS score and the total MET min/week in the whole study sample (*n* = 1220). * Spearman’s correlation coefficient.

The MDSS score in the whole study sample was 8.0 (5.0–12.0), and a total of 227 (18.6%) participants were adherent to the MD (total MDSS score ≥ 14) (Figure 1). Regarding the components of the MDSS, the highest adherence was in the consumption of potatoes (84.3%) and white meat (82.4%), while the lowest adherence was in the wine consumption (8.0%) (Table 3). Moreover, there was a significant positive correlation between the total MDSS score and the total MET min/week (r = 0.302, *p* < 0.001) (Figure 2).

**Figure 3:**
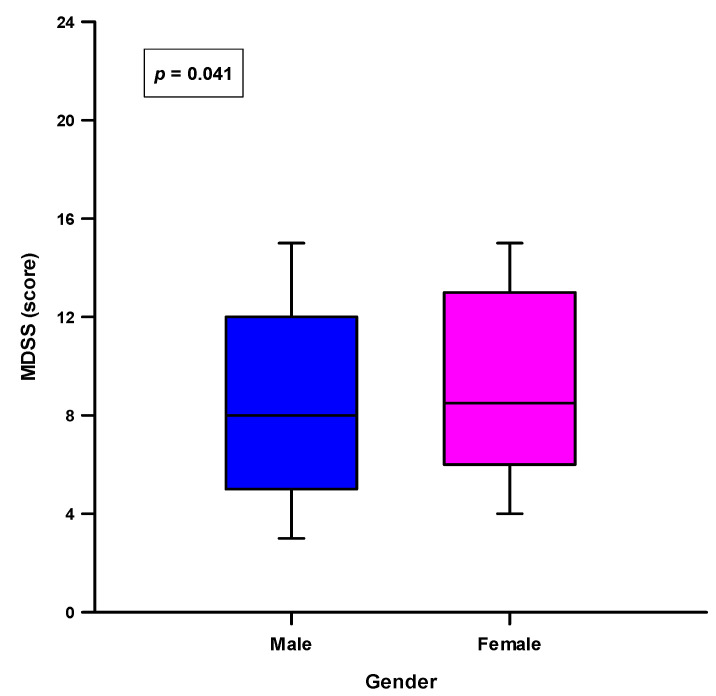
Difference of the total MDDS score between the males (*n* = 690) and females (*n* = 530). Tested with the Mann-Whitney U test.

**Figure 4:**
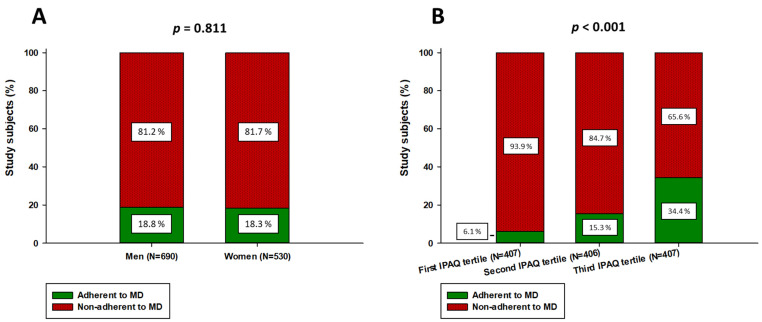
Differences in the adherence to the MD between (**A**) gender and (**B**) tertiles of the IPAQ-SF results. The chi-square test was used for analysis.

In regard to the gender differences, female participants had a significantly higher adherence in the consumption of sweets (305 (57.5%) vs. 357 (51.7%), *p* = 0.049) and fruits (161 (30.4%) vs. 165 (23.9%), *p* = 0.013). There were no significant differences regarding the adherence to the other MDSS components (Table S1). In addition, the female participants had a statistically higher total MDSS score compared to the male participants (8.5(6.0–13.0) vs. 8.0(5.0–12.0), *p* = 0.041) (Figure 3). However, when comparing the adherence to the MD (total MDSS score ≥ 14), there was no statistically significant difference between the genders (*p* = 0.811) (Figure 4).

**Figure 5:**
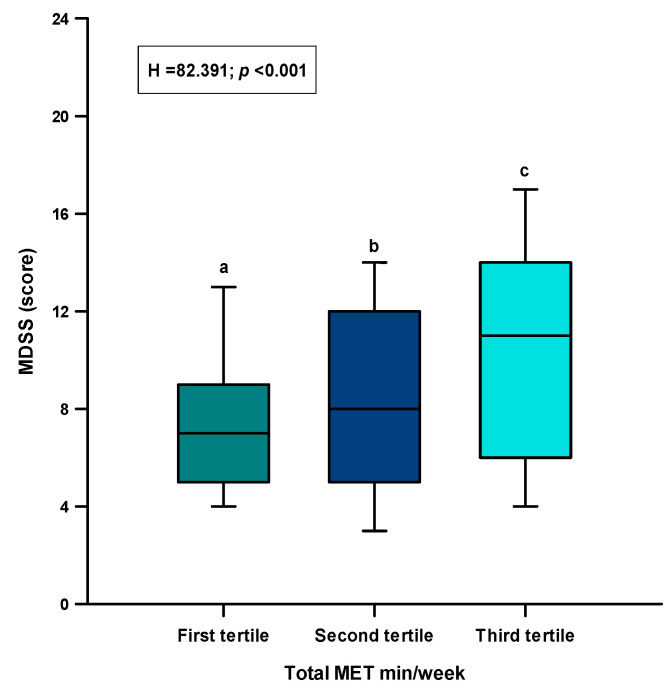
Difference of the total MDSS score between the tertiles of the IPAQ-SF results. Tested using one-way analysis of variance on ranks with the post-hoc Dunn’s test to examine the differences between each group. a vs. b = *p* < 0.05; a vs. c = *p* < 0.05; a vs. d = *p* < 0.05; d vs. b = *p* < 0.05; d vs. c = *p* < 0.05.

There were statistically significant differences in the adherence to MDSS components between the tertiles of the IPAQ-SF results (Table 3). In addition, there was a significant difference between the tertiles regarding the total MDSS score (H = 82.391, *p* < 0.001) (Figure 5). The post-hoc Dunn’s test analysis showed that there was a significant difference between all three tertiles (first tertile: 7.0 (5.0–9.0), second tertile: 8.0 (5.0–12.0), third tertile: 11.0 (6.0–14.0); *p* < 0.05) (Figure 4). Furthermore, after comparison of the adherence to the MD (total MDSS score ≥ 14), there was a significant difference between the tertiles (*p* < 0.001). Most participants who were adherent to the MD were in the third tertile (34.4%), while the least were in the first tertile (6.1%) (Figure 4).

In addition, after comparison of the durations of using a fitness center, there was a statistically significant difference between the groups (H = 13.685, *p* = 0.003) in the total MDSS score. The post-hoc Dunn’s test showed that the participants who used the fitness center for less than 1 year had the lowest total MDSS score and were significantly different (*p* <0.05) from the other three groups (<1 year: 7.0 (4.0–12.0); 1–3 years: 9.0 (6.0–12.75); 4–7 years: 8.0 (6.0–13.0); >7 years: 9.0 (6.0–12.0)).

### 3.3. Regression Analyses

Multiple linear regression analysis showed that the total MDSS score retained significant association with the total MET min/week (β ± SE, 0.007 ± 0.0006, *p* < 0.001) and the duration of using a fitness center (−0.401 ± 0.102, *p* = 0.001) after the model adjustment for age and BMI, with the total MDSS score as a dependent variable (Table 4).

Furthermore, multivariable logistic regression showed that the third and fourth quartile of MET min/week (*p* < 0.001), dietary supplements usage (*p* = 0.023) and the duration of using a fitness center for 1–3 years (*p* = 0.021) were significant predictors of positive adherence to the MD when computed along with the baseline characteristics (Table 5).

## 4. Discussion

The findings of this study showed that 18.6% of fitness center users were adherent to the MD, and there was a significant positive correlation between the level of physical activity and the MDSS score in this population. Moreover, after dividing the sample into tertiles based on the IPAQ-SF score, the third tertile (MET > 3150 min/wk) had the most fitness center users adherent to the MD, while the first tertile (MET < 1750 min/wk) had the least. With thorough research of the available literature, there are no other studies which have investigated the adherence to MD in this specific population.

Previous studies have shown that the level of fitness is significantly associated with the adherence to the MD [24,26,27,28]. A recent Spanish study on university students determined that there is a significant association between adherence to the MD and both a high level of muscular fitness and high level of cardiorespiratory fitness [28]. Furthermore, two studies conducted on schoolchildren found that subjects who were more physically active also had a higher adherence to the MD [26,27]. The biggest difference of the present study from the aforementioned ones is the studied population. Specifically, our studied population exclusively consisted of adults, which enabled more reliable inferences, as the results acquired in the pediatric population may be confounded by the effect of parental care on both dietary preferences and physical activity. Nevertheless, these results emphasize the possible connection between physical activity and the adherence to the MD. Even though the benefits of the MD are well established, studies show that the prevalence of the adherent subjects among the general population are very low, even in Mediterranean countries [35,36,37]. A study conducted on Spanish and Romanian students showed higher adherence to the MD in students who distinguished the importance of proper nutrition to achieve better health and sport results [38]. Moreover, a longitudinal study conducted on healthy adolescents showed that that the adherence to the MD significantly increased after nutrition education sessions [39]. These outcomes imply that the low adherence to the MD could be due to the lack of awareness about the benefits of this diet even if it is the traditional cuisine of the participants’ region. However, since this type of diet also minimizes meat and meat products, which are the most protein-rich type of nutrition, it is very peculiar why individuals with a high physical activity are more adherent to the MD. Our results showed that individuals with higher physical activity also use more dietary supplements, so it is possible that they meet their protein requirements through supplementation. A recent Italian study similarly showed that Mediterranean athletes used dietary supplements, but they also determined that the individuals who used supplements were already making food consumption choices that would guarantee them an adequate amount of protein intake [29]. 

After dividing the population into tertiles based on the IPAQ-SF results, there was a significant difference between these groups regarding the total MDSS score and all the distinctive dietary components of the MD. The third tertile, which had the highest physical activity, also had the highest MDSS scores, as well as the highest number of subjects adherent to the MD. In regard to specific dietary components, the third tertile also had the highest adherence to cereals, olive oil, fruits, vegetables, dairy, fish and red meat. In contrast, the first tertile, which had the lowest physical activity, also had the lowest MDSS scores, as well as the lowest number of subjects adherent to the MD. However, in regard to specific dietary components of the MD, they had the highest adherence to the potatoes, legumes, eggs and white meat. These results are interesting since the MD, as aforementioned, de-emphasizes the consumption of meat, which is usually the main source of proteins for physically active individuals. It has been well established that both professional athletes and physically active non-athletes are more prone to use white meat as a source of proteins than red meat. Knowing that better adherence to MD in terms of meat, in fact, means less intake of it, it is not surprising that participants with higher degree of physical activity were less adherent to white meat intake. This subgroup simply ingested more white meat and were thus less adherent. On the other hand, participants with higher degree of physical activity were more adherent to red meat because they ingested less red meat on average. In addition, these results could also be interpreted regarding the goals of fitness center users. Nowadays, most fitness center users are, in addition to health improvement, aiming to achieve a desired level of body composition [40]. This is especially present among adolescents and young adults, which comprised a major percentage of our study population. Furthermore, younger physically active individuals are usually also more burdened with following a healthy diet they deem fit for reaching their aims. MD would possibly be their best choice since it is currently considered one of the healthiest dietary models worldwide. However, these findings need to be addressed in future studies.

Even though there was no significant difference between genders in the number of participants adherent to the MD, the females had a significantly higher total MDSS score. This result is in line with other studies, which have shown that females are more adherent to the MD [41,42]. A study conducted on Portuguese adults showed that women have a significantly higher adherence to the MD, as well as a significantly higher number of meals per day [43]. Moreover, a recent study conducted on Italian adults showed that being female, as well as having a higher income and education level, were the most relevant factors influencing the probability of having a higher adherence to the MD [44]. Some of the possible reasons are that women tend to consume more fruit and vegetables, less meat and, in general, easily implement healthier eating patterns [45]. Another possible explanation could be that females have a tendency of being more aware about the type and quality of their diet. However, due to a specific population of our study and the different geographical distribution of the included participants, it is difficult to compare our results with these other studies. 

Out of the spotlight but also an important finding of this study is the difference regarding the total MDSS score between the different durations of using a fitness center. Our results show that participants who used the fitness center for less than 1 year also had the lowest total MDSS score compared to those who had used it for 1–3 years, 4–7 years and over 7 years. These results imply that the “newcomers” to the fitness center are significantly less adherent to the MD. However, since those who used the fitness center for longer than 1 year had significantly higher MDSS scores, we could hypothesize that, over time, the newcomers become more adherent to the MD. This could be due to their adaptation to the fitness lifestyle, which includes a more balanced and healthy diet. As aforementioned, regular fitness could possibly be associated with a healthier diet [46]. However, this finding needs to be addressed more thoroughly, especially regarding other factors which possibly influence this gradual adaptation to the MD in new fitness center users.

There are several limitations to this study. Its cross-sectional design restricts the possibility of causal conclusions. Moreover, the study was conducted in only one city in Croatia, so it is possible that these results are region-specific. Since the main tool to assess the evaluated parameters was a questionnaire, there is a possibility that the subjects had a recall bias or had an excess of subjectivity in some of the answers. Furthermore, due to the self-administration of the questionnaire, it is possible that the participants had biased or unreliable answers. Lastly, our sample mostly included a younger population, which may have interfered with the results.

## 5. Conclusions

In conclusion, this study showed that almost every fifth fitness center user in our sample was adherent to the MD. Moreover, the level of physical activity showed a significant positive correlation with the adherence to the MD. However, gender did not seem to be a strong factor in the adherence to the MD in this population. Whereas females did have a higher total MDSS score, there still was not any significant difference in the number of adherent subjects between genders. Lastly, these results showed that physical activity is also associated with dietary supplements consumption, as 82.3% of subjects in the group with the highest MET min/wk used some sort of dietary supplementation. These outcomes emphasize the importance of physical activity as they imply that, with higher levels of physical activity, people are also possibly more aware of the importance that a healthy and balanced diet has on their well-being.

## Figures and Tables

**Table 1:** Baseline characteristics of the study sample and differences regarding gender.

Parameter	Study Sample *n* = 1220	Male *n* = 690	Female *n* = 530	p *
Age (years)	29.1 ± 8.8	28.2 ± 7.8	30.3 ± 9.9	0.001
Weight (kg)	79.3 ± 15.6	87.2 ± 13.0	68.9 ± 12.1	0.001
Height (cm)	179.6 ± 9.5	184.8 ± 7.3	172.0 ± 7.4	0.001
BMI (kg/m^2^)	24.4 ± 3.4	25.4 ± 2.9	23.0 ± 3.6	0.001
Education level				
Elementary school (*n*, %)	7 (0.6)	2 (0.3%)	5 (0.9)	0.334
High school (*n*, %)	315 (25.8)	172 (24.9)	143 (27.0)	
Bachelor’s degree (*n*, %)	256 (21.0)	143 (20.7)	113 (21.3)	
Master’s degree (*n*, %)	642 (52.6)	373 (54.1)	269 (50.8)	
Using dietary supplements (*n*, %)				
Yes (*n*, %)	803 (65.8)	493 (71.4)	310 (58.5)	<0.001
No (*n*, %)	417 (34.2)	197 (28.6)	220 (41.5)	
Dietary supplements used				
Whey protein (*n*, %)	617 (76.8)	375 (76.0)	242 (78.0)	0.003
BCAA (*n*, %)	399 (49.6)	232 (47.0)	167 (53.8)	0.472
Creatine (*n*, %)	243 (30.2)	196 (39.7)	47 (15.1)	<0.001
Magnesium (*n*, %)	472 (58.7)	275 (55.7)	197 (63.5)	0.370
Vitamin C (*n*, %)	347 (43.2)	205 (41.5)	142 (45.8)	0.291
Vitamin B complex (*n*, %)	170 (21.1)	90 (18.2)	80 (25.8)	0.715
Multivitamin (*n*, %)	258 (32.1)	139 (28.1)	119 (38.3)	0.364
Duration of using a fitness center				
<1 year (*n*, %)	337 (27.6)	205 (29.7)	132 (24.9)	0.222
1–3 years (*n*, %)	352 (28.9)	187 (27.1)	165 (31.1)	
4–7 years (*n*, %)	232 (19.0)	128 (18.6)	104 (19.3)	
>7 years (*n*, %)	299 (24.5)	170 (24.6)	129 (24.3)	

All data are presented as whole numbers (percentage) or mean ± SD. Abbreviations: BMI—body mass index; MET—metabolic equivalent of task; BCAA—branched-chain amino acid. * Chi-square test or student *t*-test.

**Table 2:** Differences of the baseline characteristics between the tertiles of the IPAQ-SF results.

Parameter	First Tertile Group MET < 1750 min/Week *n* = 407	Second Tertile Group MET 1750–3150 min/Week *n* = 406	Third Tertile Group MET > 3150 min/Week *n* = 407	p *
Age (years)	29.0 ± 8.4	29.2 ± 9.3	29.1 ± 8.8	0.954
Male gender (*n*, %)	193 (47.4%)	255 (62.8%)	242 (59.5%)	<0.001
Weight (kg)	79.9 ± 15.8	77.7 ± 14.4	79.9 ± 16.2	0.095
Height (cm)	179.9 ± 9.7	179.0 ± 9.3	179.7 ± 9.5	0.473
BMI (kg/m^2^)	24.4 ± 3.3	24.0 ± 3.1	24.5 ± 3.8	0.114
Education level				
Elementary school (*n*, %)	2 (0.5)	2 (0.5)	3 (0.7)	<0.001
High school (*n*, %)	133 (32.7)	88 (21.7)	94 (23.1)	
Bachelor’s degree (*n*, %)	69 (17.0)	113 27.8)	74 (18.2)	
Master’s degree (*n*, %)	203 (49.9)	203 (50.0)	236 (58.0)	
Using dietary supplements (*n*, %)				
Yes (*n*, %)	229 (56.3)	274 (67.5)	300 (73.7)	<0.001
No (*n*, %)	178 (43.7)	132 (32.5)	107 (26.3)	
Dietary supplements used				
Whey protein (*n*, %)	115 (50.2)	206 (89.9)	296 (98.6)	<0.001
BCAA (*n*, %)	73 (31.8)	154 (67.2)	172 (57.3)	<0.001
Creatine (*n*, %)	22 (9.6)	102 (44.5)	119 (39.6)	<0.001
Magnesium (*n*, %)	144 (62.8)	165 (72.0)	163 (71.1)	0.240
Vitamin C (*n*, %)	118 (51.5)	112 (48.9)	117 (51.0)	0.893
Vitamin B complex (*n*, %)	59 (25.7)	56 (24.4)	55 (24.0)	0.916
Multivitamin (*n*, %)	84 (36.6)	90 (39.3)	84 (36.6)	0.827
Duration of using a fitness center				
<1 year (*n*, %)	98 (24.1)	118 (29.1)	121 (29.7)	<0.001
1–3 years (*n*, %)	137 (33.7)	115 (28.3)	100 (24.6)	
4–7 years (*n*, %)	76 (18.7)	94 (23.2)	62 (15.2)	
>7 years (*n*, %)	96 (23.6)	79 (19.5)	124 (30.5)	

All data are presented as whole numbers (percentage) or mean ± SD. Abbreviations: BMI—body mass index; MET—metabolic equivalent of task; BCAA—branched-chain amino acid. * Chi-square test or one-way analysis of variance.

**Table 3:** Differences in the adherence to the MDSS components between the tertiles of the IPAQ-SF results.

Parameter	First Tertile Group MET < 1750 min/Week *n* = 407	Second Tertile Group MET 1750–3150 min/Week *n* = 406	Third Tertile Group MET > 3150 min/Week *n* = 407	p *
Cereals (*n*, %)	87 (21.4)	93 (23.0)	156 (38.3)	<0.001
Potatoes (*n*, %)	367 (90.2)	343 (84.7)	318 (78.1)	<0.001
Olive oil (*n*, %)	59 (14.5)	85 (21.0)	119 (29.2)	<0.001
Nuts (*n*, %)	124 (30.5)	174 (43.0)	171 (42.0)	<0.001
Fruits (*n*, %)	70 (17.2)	98 (24.1)	158 (38.8)	<0.001
Vegetables (*n*, %)	91 (22.4)	111 (27.3)	178 (43.7)	<0.001
Dairy (*n*, %)	64 (15.7)	118 (29.1)	154 (37.8)	<0.001
Legumes (*n*, %)	313 (76.9)	248 (61.2)	270 (66.3)	<0.001
Eggs (*n*, %)	224 (55.0)	186 (46.0)	195 (47.9)	0.025
Fish (*n*, %)	242 (59.5)	217 (53.7)	266 (65.4)	0.003
White meat (*n*, %)	372 (91.4)	304 (74.9)	329 (80.8)	<0.001
Red meat (*n*, %)	73 (17.9)	145 (35.8)	187 (45.9)	<0.001
Sweets (*n*, %)	206 (50.6)	265 (65.3)	191 (46.9)	<0.001
Wine (*n*, %)	1 (0.2)	59 (14.6)	38 (9.3)	<0.001

All data are presented as whole numbers (percentage). * Chi-square test.

**Table 4:** Multiple linear regression model of the independent predictors of the total MDSS score.

Variable.	β *	SE	t-Value	*p*
Age	−0.003	0.013	−0.222	0.824
BMI	0.017	0.038	0.464	0.642
Total MET min/week	0.007	0.0006	11.509	<0.001
Duration of using a fitness center	0.401	0.102	3.912	0.001

Abbreviations: SE—standard error; BMI—body mass index; MET—metabolic equivalent of task. * unstandardized coefficient β.

**Table 5:** Multivariable logistic regression analysis of the independent predictors for positive adherence to the MD according to the total MDSS score.

Variable	aOR [95% CI]	*p*
Female sex ^1^	1.09 [0.77, 1.54]	0.811
Using the fitness center for 1–3 years ^2^	1.93 [1.24, 3.00]	0.021
Using the fitness center for 4–7 years ^2^	1.47 [0.97, 2.23]	0.071
Using the fitness center for >7 years ^2^	1.05 [0.68, 1.63]	0.814
Dietary supplements usage ^3^	1.52 [1.06, 2.17]	0.023
Older age	1.00 [0.98, 1.02]	0.874
Total MET min/week 2nd quartile ^4^	1.61 [0.88, 2.96]	0.126
Total MET min/week 3rd quartile ^4^	3.94 [2.28, 6.80]	<0.001
Total MET min/week 4th quartile ^4^	8.08 [4.78, 13.67]	<0.001
BMI	0.99 [0.95, 1.05]	0.936

^1^ Reference group are male subjects. ^2^ Reference group are subjects with the shortest gym attendance (<1 year). ^3^ Reference group are subjects not utilizing dietary supplements. ^4^ Reference group are subjects within the 1st MET quartile. Abbreviations: MD—Mediterranean diet; OR—multivariable adjusted odds ratio; 95% CI—95% confidence interval; BMI—body mass index; MET—metabolic equivalent of task.

## Data Availability

All the data is available upon request to the corresponding author.

## Supplementary Materials

The following are available online at https://www.mdpi.com/article/10.3390/nu13114038/s1, Table S1: Adherance to the MDSS components and differences regarding gender.
