# Supplementary material for: Adherence to the Mediterranean Diet and Its Association with the Level of Physical Activity in Fitness Center Users: Croatian-Based Study
Source: Nutrients. 2021 Nov 12;13(11):4038. doi: 10.3390/nu13114038 (PMC8625296; doi:10.3390/nu13114038)
Supplement: Supplementary file 1 [file nutrients-13-04038-s001.zip › nutrients-1454364-supplementary.pdf]

Supplementary Material

Table S1. Adherance to the MDSS components and differences regarding gender.

Parameter	Study Sample N = 1220	Males N = 690	Females N = 530	*p* *
Cereals (N, %)	336 (27.6)	186 (27.0)	150 (28.3)	0.659
Potatoes (N, %)	1028 (84.3)	579 (83.9)	449 (84.9)	0.704
Olive oil (N, %)	263 (21.6)	146 (21.2)	117 (22.2)	0.726
Nuts (N, %)	469 (38.5)	275 (39.9)	194 (36.7)	0.283
Fruits (N, %)	326 (26.7)	165 (23.9)	161 (30.4)	0.013
Vegetables (N, %)	380 (31.1)	203 (29.4)	177 (33.4)	0.154
Dairy (N, %)	336 (27.6)	185 (26.8)	151 (28.5)	0.544
Legumes (N, %)	831 (68.2)	462 (67.0)	369 (69.8)	0.328
Eggs (N, %)	605 (49.7)	339 (49.2)	266 (50.3)	0.751
Fish (N, %)	725 (59.5)	405 (58.8)	320 (60.5)	0.586
White meat (N, %)	1005 (82.4)	561 (81.3)	444 (83.8)	0.295
Red meat (N, %)	405 (33.2)	225 (32.7)	180 (34.0)	0.675
Sweets (N, %)	662 (54.3)	357 (51.7)	305 (57.5)	0.049
Wine (N, %)	98 (8.0)	58 (8.4)	40 (7.6)	0.666

All data are presented as whole numbers (percentage). * Chi-square test.
